# Scheduled and Breakthrough Opioid Use for Cancer Pain in an Inpatient Setting at a Tertiary Cancer Hospital

**DOI:** 10.3390/curroncol31030101

**Published:** 2024-03-05

**Authors:** Aline Rozman de Moraes, Elif Erdogan, Ahsan Azhar, Suresh K. Reddy, Zhanni Lu, Joshua A. Geller, David Mill Graves, Michal J. Kubiak, Janet L. Williams, Jimin Wu, Eduardo Bruera, Sriram Yennurajalingam

**Affiliations:** 1 Department of Palliative Care, Rehabilitation Medicine, and Integrative Medicine, UT MD Anderson Cancer Center, Houston, TX 77030, USA; arozman@mdanderson.org (A.R.d.M.); elifongelmd@gmail.com (E.E.);; 2 Department of Biostatistics, UT MD Anderson Cancer Center, Houston, TX 77030, USA

**Keywords:** cancer pain, hospitalized patients, breakthrough opioids, daily opioid dose

## Abstract

**Background**: Our aim was to examine the frequency and prescription pattern of breakthrough (BTO) and scheduled (SCH) opioids and their ratio (BTO/SCH ratio) of use, prior to and after referral to an inpatient supportive care consult (SCC) for cancer pain management (CPM). **Methods and Materials**: Patients admitted at the MD Anderson Cancer Center and referred to a SCC were retrospectively reviewed. Cancer patients receiving SCH and BTO opioids for ≥24 h were eligible for inclusion. Patient demographics and clinical characteristics, including the type and route of SCH and BTO opioids, daily opioid doses (MEDDs) of SCH and BTO, and BTO/SCH ratios were reviewed in patients seen prior to a SCC (pre-SCC) and during a SCC. A normal BTO ratio was defined as 0.5–0.2. **Results**: A total of 665/728 (91%) patients were evaluable. Median pain scores (*p* < 0.001), BTO MEDDs (*p* < 0.001), scheduled opioid MEDDs (*p* < 0.0001), and total MEDDs (*p* < 0.0001) were higher, but the median number of BTO doses was fewer (2 vs. 4, *p* < 0.001), among patients seen at SCC compared to pre-SCC. A BTO/SCH ratio over the recommended ratio (>0.2) was seen in 37.5% of patients. The BTO/SCH ratios in the pre-SCC and SCC groups were 0.10 (0.04, 0.21) and 0.17 (0.10, 0.30), respectively, *p* < 0.001. Hydromorphone and Morphine were the most common BTO and SCH opioids prescribed, respectively. Patients in the early supportive care group had higher pain scores and MEDDs. **Conclusions**: BTO/SCH ratios are frequently prescribed higher than the recommended dose. Daily pain scores, BTO MEDDs, scheduled opioid MEDDs, and total MEDDs were higher among the SCC group than the pre-SCC group, but the number of BTO doses/day was lower.

## 1. Introduction

Pain is a frequent and distressing symptom among cancer patients [1,2]. Pain in these patients may be due to their disease, its treatment, or nonmalignant causes. Approximately 70–80% of cancer patients will have cancer-related pain, and this prevalence is even higher in advanced cancer patients. Despite recent advances in cancer pain management (CPM), cancer pain is not controlled [3,4,5]. Pain interferes with patients’ work, mood, and enjoyment of life. Despite significant progress in CPM over the last few decades, recent studies indicate unsatisfactory outcomes due to poor assessments and suboptimal treatments [6]. Most importantly, uncontrolled pain remains the most common reason for inpatient supportive/palliative care consultation [7,8,9]. Background pain is a continuous and constant pain present at rest [10]. Breakthrough pain is a transitory increase of pain in cancer patients with adequately controlled background pain [10]. Breakthrough pain’s prevalence is nearly 50%, and is usually defined with a rapid onset, a short duration, and severe intensity, and averages four episodes a day [9,10,11,12]. Presence of breakthrough pain has been considered as a negative prognostic factor that interferes with quality of life of patients [9,12,13,14,15]. For effective pain management, the cancer pain guidelines recommend pain screening, comprehensive assessment, and monitoring of pain after the start of pain treatments [16]. Pain is often a complex multidimensional experience, consisting of sensory and affective dimensions. Cancer pain can be regarded as “total pain”, consisting of physical, psychological, spiritual, and social dimensions. Often, standardized and validated assessment tools that can be routinely used in clinical practice are used to assess pain. Some commonly used assessment tools used are the 0–10 numeric rating scale or visual analog scale. The Edmonton symptom assessment scale (ESAS) is frequently used by supportive care clinicians as it not only assesses pain but other symptoms related to pain, such as depression, anxiety, or drowsiness, and thereby helps to assess the ‘total pain’, and its serial assessment may provide an overall picture of pain control during an inpatient hospital admission. In addition to assessment of the severity of pain, assessment of other ‘pain characteristics’ is important, which usually includes the type of pain, both nociceptive (somatic, visceral) and neuropathic, the presence of non-opioid risk behavior, and delirium. In an ambulatory setting, pain assessment can be accomplished using pain dairy’s or, more recently, using digital health applications, such as mobile apps for pain assessment. These mobile phone apps help in regular pain assessments, provide timely feedback to patients and their clinicians, facilitate patient education, and help physicians to make timely medication changes and improve patient–physician communication [17]. However, there are still challenges which prevent the routine use of mobile phone apps. Some of the main barriers are socioeconomic status, data protections, and evidence-based app validation [18].

As opioids are the primary treatment for moderate-to-severe cancer pain, successful CPM involves the use of effective opioid doses (both scheduled and breakthrough or rescue opioid doses) and is an indicator of end-of-life quality of care in palliative care. An adequate treatment of breakthrough pain is important because it is associated with significant anxiety, depression, and low quality of life [12,14,19,20,21,22]. Traditionally, scheduled long-acting opioids are used for background pain and short-acting immediate-release oral opioids are used on an “as needed” basis to treat breakthrough pain [13,23,24,25]. There are different types of short- and long-acting opioids, such as Morphine, Hydromorphone, Oxycodone, Methadone, Oxymorphone, and Transdermal and transmucosal Fentanyl, for scheduled and breakthrough pain [24,26,27]. In hospitalized cancer patients, the use and ratios of scheduled (SCH) and breakthrough pain or rescue (BTO) opioids have a very wide range [24,27,28]. Scheduled opioids have no ceiling effect and therefore no dosing limit may be required until they are associated with unmanageable side effects, like constipation, confusion, delirium. The dose of BTO opioids used is proportionate to the opioid daily dose [7]. The current recommendation for BTO dosage ranges from 5–20% of the daily opioid dose every one or two hours, as required. In the elderly, BTO dosage is usually a lower percentage (5%) of the total daily opioid dose every 4 h, as required [29,30,31,32,33].

However, the use of scheduled and BTO opioids and their ratio in routine clinical practice in inpatient cancer patients is not clear, especially regarding supportive/palliative care vs. oncology care teams. This information may be helpful to determine strategies to better control cancer pain, especially breakthrough pain, and minimize the side-effects of opioids. Our aim was to examine the frequency and prescription pattern of BTO and scheduled opioids and their ratio (BTO/SCH ratio) of use prior to and after referral to an inpatient supportive care consult (SCC) for CPM.

## 2. Materials and Methods

This retrospective study was approved by the Institutional Review Board of MD Anderson, which waived the requirement for informed patient consent.

For the conduct of this study, the goal of which was to capture the opioid prescription use of scheduled and breakthrough opioids in routine clinical practice in a tertiary cancer setting, we have used a retrospective design due to its ability to analyze long-term trends through existing medical records. This design was chosen to capture the real-time practice patterns in cancer hospital settings. The retrospective design was also chosen as it was more feasible to obtain the outcomes aimed for in our study than a prospective design due to resources, time, and expense [34].

Patients admitted in the inpatient setting at the MD Anderson Cancer Center who had a SCC between 1 June 2017 and 31 May 2018 were reviewed retrospectively. Patients were eligible if they were older than 18 years old and receiving opioids for at least 24 h.

SQUIRE guidelines were used to ensure completeness and transparency [35].

### 2.1. Process of the Supportive Care Service and Decision-Making Process for Opioid Prescription Adjustments

At The University of Texas M. D. Anderson Cancer Center, Houston, USA, a 698-bed comprehensive cancer center, inpatient supportive care consultations have been available since October 1999. The full-time supportive care team consists of board-certified palliative care specialists, palliative care and oncology fellows, advanced practice providers, pharmacists, chaplains, social workers, case managers, psychologists, and counselors. The program provides symptom control and palliative care in all areas of the cancer center by consultation or mobile team on daily basis. In addition, the program includes an outpatient supportive care clinic and an acute palliative and supportive care unit, wherein patients with distressing symptoms are admitted for control of their symptoms and for help in transitioning home or to hospice care. The mobile team comprises the above team members, except that social workers and case managers vary based on the type of cancer, e.g., patients with genitourinary cancers have a separate social worker and case manager from the ones for patients with lung cancer. The team’s primary focus is on pain, symptom control, palliative care, and end-of-life issues. The care of all patients follows a standardized management plan [36,37,38]. Patients and their families are initially assessed by palliative care, oncology fellows, or advanced practice providers, using tools such as the ESAS [39], Memorial Delirium Assessment Scale (MDAS) [40], and constipation and family support questionnaires. The findings are then discussed with a palliative care specialist, who then conducts an interview with the patient and the family and performs a physical examination. The physician then requests appropriate members of the interdisciplinary team to participate based on the patient’s and family’s individual needs. These interventions and care provided by the interdisciplinary team follow PC guidelines established by the National Comprehensive Cancer Network and National Consensus Project and have been outlined elsewhere. These guidelines focus on (a) assessing and managing cancer-related symptoms, including pain, fatigue, anorexia, anxiety, depression, sedation, dyspnea, sleep disturbance, and impaired feeling of wellbeing; (b) providing assistance to the patient’s and caregivers’ understanding of the disease and treatment goals; and (c) providing assistance to the patient and their caregivers in coping with life-threatening illness and in decision-making.

Opioids are the primary treatment for cancer pain management. The decision-making process for opioid prescription adjustments is made after the patient assessment described above, which includes assessing pain and symptoms using the ESAS and MDAS. When patients present with poor pain control, opioid-induced side effects such as nausea and constipation, opioid-induced neurotoxicity such as myoclonus, hallucinations, or if the current route or formulation is no longer feasible, the opioids are either replaced by another (opioid switching or opioid rotation), route changed (e.g., change from oral route to intravenous), or dose changed (increased). The patient is always prescribed a scheduled opioid to control the background pain, and breakthrough doses, usually at 1–4 h intervals, to control breakthrough pain episodes inherent with cancer pain. The dosage for breakthrough opioids is usually 10–15% of the scheduled dosing. Following a changed pain prescription, the patient is monitored daily with assessments as done at the initial visit, and medications are adjusted until hospital discharge by the supportive care team. Most importantly, once the supportive care team is involved in the pain management of a given referred patient, the responsibility of the patient’s pain control and prescription of opioids is solely under their control and no changes are made, either by the primary oncology team responsible for the patient’s care in the hospital or the other teams involved in the patient’s care, without consulting the supportive care team. In addition to opioids, other treatments for cancer pain management are provided based on the assessment. This may include the use of non-steroidal anti-inflammatory medications, steroids, antidepressants such as duloxetine, referral for physical therapy, counseling, chaplaincy, radiation therapy, or procedures such as neurolytic blocks, cordotomy, etc.

Data prior to and after a SCC referral after 72 h of hospital admission were collected. The data obtained prior to the referral to a SCC were termed as the “pre-supportive care group”, and the data from after referral as the “supportive care group (after referral to SCC)”, respectively. In the pre-supportive care group, cancer pain was managed by the primary oncology care team prior to their referral to a SCC. Patients that were referred to a SCC within 72 h of their admission were classified as the “early supportive care group”. The rationale for the use of a pre-supportive care group and supportive care group was to understand patient characteristics, pain, and opioid prescription use for scheduled and breakthrough opioids after the systematic assessment and management by a specialized supportive care team. The early supportive care cohort was evaluated to assess patient characteristics, pain, and opioid prescription use after the systematic assessment and management by a specialized pain/supportive care team at the time of hospital admission rather than later (after 72 h of admission).

### 2.2. Assessments

Demographics and clinical information (performance status, cancer diagnosis, relationship between SCH and BT opioids, the type, route, and frequency of administration of SCH and BT opioids, and dosage, assessed using Morphine Equivalent Daily Dose (MEDD) scores, Edmonton symptom assessment scale (ESAS) scores, CAGE-AID questionnaire scores, and Memorial delirium assessment scale (MDAS) scores), were collected for all enrolled patients.

ESAS, CAGE-AID, and MDAS scores are part of the standard of care in SCCs and ambulatory clinical visits.

**ESAS** is a tool designed by our group to assist in the assessment of ten symptoms common in cancer patients over the prior 24 h: pain, fatigue, nausea, depression, anxiety, drowsiness, shortness of breath, appetite, sleep, and feelings of well-being [38]. The severity at the time of assessment of each symptom is rated from 0 to 10 on a numerical scale, 0 meaning that symptom is absent and 10 meaning that it is of the worst possible severity. A higher score indicates higher symptom intensity. The instrument is both valid and reliable in the assessment of the intensity of symptoms in cancer populations. All the assessments were completed by the patients themselves, or with the help nurse or a caregiver. The pain item of ESAS is the one used to assess pain in the inpatient setting of a cancer hospital. The optimal cutoffs were ≥1 point on the ESAS pain item for improvement of pain, and ≤1 point for deterioration of pain [41].

The **CAGE-AID (cut down, annoyed, guilty, eye opener)** questionnaire is a four-item validated tool that is used to screen for a history of alcoholism or drug abuse and the presence of severe symptom distress and potential non-medical opioid use in cancer patients. A score ≥ 2 was considered positive and has been reported to be more than 85% sensitive and 90% specific for the diagnosis of alcohol/drug abuse and/or dependence [42].

The **MDAS** is structured as a ten-item, four-point clinician-rated scale designed to qualify the severity of delirium in medically ill patients [40]. Items included in the MDAS reflect the diagnostic criteria for delirium in the DSM IV. Scale items assess the disturbances in arousal and level of consciousness, as well as several areas of cognitive functioning (memory, attention, orientation, and disturbances in thinking) and psychomotor activity. The MDAS yields a global score ranging from 0 to 30, with a suggested cut-off score of 7 for delirium.

Study data were collected and managed using Research Electronic Data Capture (REDCap) electronic data capture tools hosted at the MD Anderson Cancer Center [43,44]. REDCap is a secure, web-based software platform designed to support data capture for research studies, providing (1) an intuitive interface for validated data capture; (2) audit trails for tracking data manipulation and export procedures; (3) automated export procedures for seamless data downloads to common statistical packages; and (4) procedures for data integration and interoperability with external sources. All data were collected for trained researchers.

### 2.3. Statistical Analysis

Data were summarized using standard descriptive statistics such as mean, standard deviation, median and range for continuous variables, and frequency and proportion for categorical variables. Association between categorical variables was examined by the Chi-squared test, Fisher’s exact test, or McNemar’s test when appropriate. Differences in continuous variables before and after referral to supportive care were compared using the Wilcoxon signed-rank test. For the comparison between early (before 72 h of hospitalization) and late referral (after 72 h of hospitalization) supportive care groups, the Wilcoxon rank-sum test was used to examine the difference of continuous variables between groups.

Sample size calculation: For the primary objective, we compared MEDD scores for scheduled pain control and breakthrough pain control before (pre-supportive care) and after (supportive care) referral after 72 h. With 364 patients, we would have a 90% power to detect an effect size of 0.185 in MEDD score difference for scheduled pain control and for breakthrough pain control using a paired *t*-test with a 2-sided type I error rate of 0.025.

All computations were carried out in SAS 9.4 (SAS Institute Inc., Cary, NC, USA).

## 3. Results

**Figure 1:**
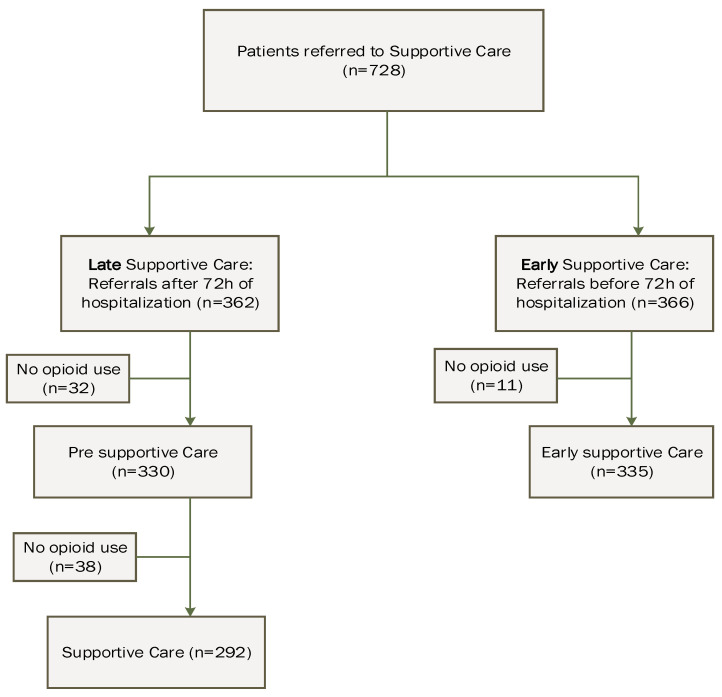
Study flow diagram. Pre-supportive care: data from patients referred after 72 hours (h) of hospitalization and before supportive consult (pain management by treating oncology service); supportive care: data from patients referred after 72 h of hospitalization and after supportive consult; early supportive care: data from patients referred to supportive care before 72 h of hospitalization and before.

Figure 1 shows the study flow diagram. In total, 665/728 (91%) patients were evaluable. Of these, 362 patients were referred to the supportive care service after 72 h of hospitalization, and data were compared before (pre-supportive care, *n* = 330) and after (*n* = 292) referral to supportive care. Due to the absence of opioid use, 32 and 38 patients were excluded from analysis from the pre-supportive care and supportive care groups, respectively. A total of 355 of the 366 patients referred to the supportive care service before 72 h of hospitalization (early supportive care) were analyzed. Eleven patients were excluded due to the absence of opioid use.

Patients in the early supportive care group were younger (*p* = 0.0018), female (*p* = 0.09), had higher illicit drug use (*p* = 0.016), higher pain scores (*p* = 0.007), higher MEDDs (*p* < 0.001), and higher BTO MEDDs (*p* = 0.018). When patients referred earlier (early supportive care group) and later (supportive care group), Hydromorphone (36.3% in the supportive care group vs. 42.6% in the early supportive care group, *p* = 0.26) and Morphine (33.3% in the supportive care group vs. 32.8% in the early supportive care group, *p* = 0.30) were the most common BTO opioids prescribed in both groups. Morphine was the most commonly prescribed SCH opioid in both groups (36% in the supportive care group and 38.1% in the early supportive care group, *p* = 0.30). A BTO/SCH relation over the recommended ratio (>0.2) was seen in 490 patients (51%).

The clinical and demographic characteristics of patients in the pre-supportive care and supportive care groups are summarized in Table 1. There were no significant differences in age, gender, marital status, cancer diagnosis, race, frequency of smoking status, illicit drug use, CAGE positive scores, MDAS scores, and number of follow up visits between the pre-supportive and supportive care groups. Pain scores were higher among the patients in the supportive care group both at initial visit (*p* < 0.001) and follow up (*p* < 0.001) when compared to the pre-supportive care group. The pain change from initial visit at follow up visit was −1 points on the ESAS 0–10 scale, which is equivalent to the minimal clinically important difference for the ESAS Pain item. The median number of BTO doses was lower in the supportive care group, 2 vs. 4 (*p* < 0.001). BTO MEDDs (*p* < 0.0001), scheduled opioid MEDDs (*p* < 0.0001), and total MEDDs (*p* < 0.0001) were higher among the patients in the supportive care group.

**Figure 2:**
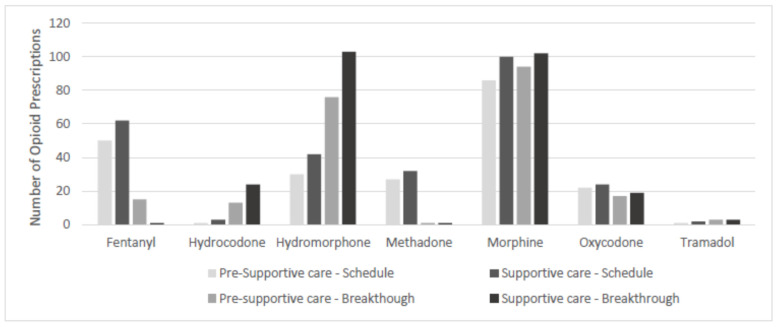
Opioid prescription at Pre-Supportive Care and Supportive Care. Medication prescription per patient. Codeine and Oxymorphone are not shown due to low N. No statistically significant differences between groups (McNemar’s test); pre-supportive care: data from patients referred after 72 h of hospitalization and before supportive consult; supportive care: data from patients referred after 72 h of hospitalization and after supportive consult.

Hydromorphone (34.7% in the pre-supportive care group vs. 40.7% in the supportive care group) and Morphine (42.9% in the pre-supportive care group vs. 40.71% in the supportive care group) were the most common BTO opioids prescribed in both groups (Figure 2). Morphine (39.6% in the pre-supportive care group vs. 37.7% in the supportive care group) and Fentanyl (23% pre-supportive care vs. 23.4% supportive care) were the most common scheduled opioids prescribed in both groups.

The BTO/SCH ratio ranged from 0.07 to 0.30, with higher values for the supportive care group (Table 2). BTO/SCH ratios over the recommended ratio (>0.2) were seen in 192 patients (37.5%) (Table 2). Comparisons (median, IQR, *p*-values) for the BTO/SCH ratios for the pre-supportive care group and supportive care group were 0.10 (0.04, 0.21) and 0.17 (0.10, 0.30), *p <* 0.001 (Table 2). The ratio for hydromorphone in the pre-supportive care group was 0.63 (0.03, 1.25), and in the supportive care group was 0.12 (0.09, 0.18) (Table 3).

## 4. Discussion

There are limited studies investigating the scheduled and breakthrough opioid use patterns for cancer pain management by inpatient supportive care teams. In this study, we found that patients receiving cancer pain management from supportive care, when compared to prior to supportive care consultation (pre-supportive care group), had higher pain scores, MEDDs, and BTO daily doses. The supportive care group had lower numbers of BTO doses used and improved pain scores at follow-up visits than the pre-supportive group. These results may suggest better opioid prescription use, but further studies are needed to validate these findings. Most of the patients in the pre-supportive and supportive care groups received higher-than-recommended BTO/SCH ratios. Patients in the early supportive care group had higher pain scores and daily opioid use (MEDDs), and higher BTO MEDDs, suggesting higher levels of distress and supportive care needs. Even though it is a retrospective study, this is an interesting paper as it reports the routine daily practice of opioid pain management at one of the main tertiary cancer centers in the United States of America.

In a study published by Mercadante et al. (2010), Morphine and oral transmucosal Fentanyl were the most common BTO opioids prescribed among patients admitted to an acute palliative care unit, with Morphine being prescribed for 386 episodes of breakthrough pain and oral transmucosal Fentanyl for 152 episodes [45]. Qian et al. (2020) showed that Morphine (38%), Hydromorphone (17%), and Fentanyl (15%) were the most common SCH opioids prescribed between patients hospitalized and receiving palliative care consultation [46]. Mercadante et al. (2017) found that sublingual Fentanyl for the treatment of breakthrough pain was associated with safe and effective analgesia in cancer patients receiving low doses of SCH opioids [47]. In another study by Mercadante et al. (2020), cancer patients on lower doses of SCH opioids (MEDD < 60 mg/day) had fewer episodes of breakthrough pain, with less severe pain intensity and early pain onset pain, as well as longer times to meaningful pain relief taking breakthrough opioids and less satisfaction with BTO [48]. Currow et al. (2020) found that oral Morphine was ineffective as a BTO treatment at ratios of 0.16, 0.12, and 0.08 for the effective management of breakthrough pain and was at different dose ranges proportional to the SCH opioid dose [49]. Azhar et al. (2019) found that BTO doses were 10% of scheduled opioid doses [29]. However, results reported were based on patient satisfaction rather than actual pain scores. In our study, the median ratio was over 0.15 in all groups and more than 37% of patients had ratios that were over 0.2 [49,50,51,52]. The ratios were higher when Hydromorphone or Morphine were used as a SCH medication.

Compared to patients referred to supportive care after 72 h, patients in the early supportive care group were younger females, having higher levels of pain and BTO daily doses. However, the BTO/SCH ratio for Hydromorphone, the most common opioid in patients referred to supportive care after 72 h, was 0.73, and was 0.23 in the early supportive group. These findings suggest that the early supportive care group was associated with a higher risk for poor pain control and higher opioid dose needs for breakthrough pain. Further, in a study conducted by Azhar et al. (2019), young age and higher ESAS scores for pain had a significant association with poor responses to immediate-release opioids for breakthrough pain, which could explain why the total and BTO MEDDs were higher among the patients that were referred earlier to supportive care [29]. Further studies need to be done to study whether there is an association between BTO/SCH ratio and better pain outcomes such as pain improvement, attainment of personalized pain goals, non-medical opioid use, and toxicity.

Future studies are needed to optimize the use of opioid (scheduled and breakthrough) prescriptions to improve pain control and thereby patients’ overall quality of life. Some of the interventions to consider include the use of a patient’s genetic data, such as single nucleotide polymorphisms of candidate genes such as inflammatory genes, to determine the sensitivity to a particular opioid type for a given patient, the dose needed for a opioid to be effective, and the risk of opioid-related side effects [53]. In a recent study by our team, we assessed the genetic factors associated with pain severity, daily opioid dose, and pain response to opioids. The results of the study suggest single nucleotide polymorphisms of OPRM1, COMT, NFKBIA, CXCL8, IL-6, STAT6, and ARRB2 genes were significantly associated with pain severity, opioid daily dose, and pain response [53]. Similar findings were found in other studies investigating the use of pharmacogenomics for personalized pain management [54]. Further studies are needed.

In recent years, there has been increased use of Artificial Intelligence (AI) to provide patient care, as well as in pain research. AI may have the potential to provide better pain control, as in traditional pain assessment and management methods there is a high likelihood of variability of patient-reported pain scores, the perception of pain by different individuals, and the algorithms for pain management which includes the use of opioids and other pain interventions. AI technologies such as machine learning, deep learning, and natural language processing have been used for pain assessment, surveillance and monitoring, and opioid misuse risk prediction. However, there is limited published research on the use of AI for pain management [54,55,56,57]. Future studies are needed on patients with cancer pain using AI, and these should utilize the recent advances in pain assessments such as facial image analysis [54]. Better cancer pain management may be facilitated by using predictive clinical decision systems which incorporate patients’ clinical data, patient data obtained from wearable devices which assess pain, sleep, and activity, and biomarkers as discussed above, such as single nucleotide polymorphisms or various candidate genes which may predict the sensitivity to certain opioids, response rates of a given pain type to an opioid, or other pain treatment. However, the use of AI may only supplement clinician decision-making processes for the management of cancer pain rather than replace them, due to their inherent limitations.

Our study has several limitations. It is a single-center study and therefore some of the results may not be generalizable to other cancer and community hospital settings. The data used in our study were obtained prior to the COVID-19 pandemic; however, recent research suggests that the issues discussed in our study in regards to SCH and BTO opioid use in cancer patients would be still applicable to the current economic and social landscape, based on recently published studies [50,51,52,58]. A further limitation of this study was that our study found a lower number of BTO doses in the supportive care group but was unable to capture whether the opioid prescription changes made by the supportive care group had any impact on their breakthrough pain episodes. Further studies are needed.

## 5. Conclusions

BTO/SCH ratios were frequently prescribed higher than the recommended dose. Daily pain scores, BTO MEDDs, scheduled opioid MEDDs, and total MEDDs were higher among patients seen at a supportive care group, but their number of BTO doses/day was smaller. Further studies are needed.

## Figures and Tables

**Table 1:** Demographic & Clinical Characteristics.

	Pre-Supportive Care (*n* = 330)	Supportive Care (*n* = 292)	*p* *
Age in years, (median IQR)	60 (52, 70)	60 (52, 70)	0.99
Female, *n* (%)	160 (48.5%)	146 (49.8%)	0.59
Cancer diagnosis, *n* (%)			0.09
Gastrointestinal	96 (29.1%)	90 (30.6%)	
Genitourinary	30 (10.3%)	32 (10.9%)	
Hematologic	60 (18.2%)	50 (17.1%)	
Lung	41 (12.4%)	37 (12.6%)	
Gynecological	33 (10%)	30 (10.2%)	
Head and neck	22 (6.7%)	18 (6.1%)	
Breast	23 (7%)	19 (6.5%)	
Marital Status, *n* (%)			0.21
Divorced/legally separated	32 (9.7)	30 (10.2%)	
Married/significant other	225 (68.4%)	194 (66.2%)	
Single	44 (13.4%)	40 (13.7%)	
Widowed	23 (7%)	23 (7.9%)	
Race, *n* (%)			0.998
White or Caucasian	223 (67.6%)	199 (68.2%)	
Black or African American	43 (13.1%)	39 (13.4%)	
Asian	20 (6.1%)	16 (5.5%)	
Smoking Status, *n* (%)			0.853
Current	19 (5.8%)	17 (5.8%)	
Former	109 (33%)	101 (34.5%)	
Never	170 (51.5%)	149 (50.8)	
Illicit Drug Use			0.59
Yes	18 (5.9%)	17 (6.3%)	
Number of follow up visits	2 (1, 5)	2 (1, 5)	1.0
ECOG, *n* (%)			<0.001
≤2	75 (56.8%)	68 (58.2%)	
3 or 4	57 (43.2%)	49 (41.8%)	
CAGE-AID, *n* (%)			1.0
Positive (≥2)	13 (5.5%)	13 (6%)	
MDAS **, median (IQR)	1 (0, 4)	1 (0, 4)	1.0
Pain score (0–10), median (IQR) at baseline	4 (1, 7)	6 (3, 6)	<0.001
Follow up pain, median (IQR)	6 (4, 8)	5 (3, 7)	<0.001
Difference (follow up-baseline), median (IQR)	1 (−1, 4)	−1 (−3, 0)	<0.001
Number of doses of BTO, median (IQR)	4 (2, 8)	2 (1, 4)	<0.001
Number of Schedule doses median (IQR)	4 (2, 9)	2 (1, 6.5)	0.07
BTO MEDD ***, median (IQR)	11.46 (4.5, 26.25)	20 (10, 50)	<0.0001
Scheduled MEDD ***, median (IQR)	0 (0, 12.5)	9.38 (0, 50)	<0.0001
Total MEDD ***, median (IQR)	18.5 (6.67, 45.26)	44.38 (17.5, 90)	<0.0001

* Categorical variables examined by McNemar’s test; continuous variable examined by Wilcoxon signed-rank-sum test; ** MDAS: Memorial delirium assessment scale; *** MEDD: morphine equivalent daily dose.

**Table 2:** Ratio of MEDD Breakthrough/MEDD Schedule opioids.

	Pre-Supportive Care (*n =* 330)	Supportive Care (*n* = 292)	p *
Ratio of MEDD BTO/MEDD Schedule Opioids, median IQR	0.10 (0.04, 0.21)	0.17 (0.10, 0.30)	<0.001 *
Under, *n* (%)	121 (49%)	51 (19.2%)	0.69
Normal, *n* (%)	57 (23.1%)	91 (34.3%)	
Over, *n* (%)	69 (27.9%)	123 (46.4%)	

Abbreviations: MEDD: morphine equivalent daily dose. IQR: Inter quartile range. BTO: breakthrough opioids. * Continuous variable examined by Wilcoxon signed-rank-sum test; categorical paired variables were evaluated by McNemar’s test. Ratio between opioids classified as under if BTO/Schedule MEDD ratio ≤ 0.1, normal if the BTO/Schedule MEDD ratio > 0.1 and ≤0.2, or over if the BTO/Schedule MEDD ratio > 0.2.

**Table 3:** Ratio of MEDD Breakthrough/Scheduled opioid per medication.

	Breakthrough	Pre-Supportive Care		Supportive Care		
		Hydromorphone (N = 76)	Morphine (N = 94)	Hydromorphone (N = 103)	Morphine (N = 102)	
		Median (IQR)	Median (IQR)	Median (IQR)	Median (IQR)	*p* *
**Scheduled**	Fentanyl	0.06 (0.04, 0.10)	0.10 (0.07, 0.21)	0.13 (0.10, 0.21)	0.21(0.10, 0.42)	0.043
	Hydromorphone	0.63 (0.03, 1.25)	0.09 (0.05, 1.28)	0.12 (0.09, 0.18)	NA	0.33
	Methadone	0.04 (0.03, 0.09)	0.03 (0.01, 0.22)	0.20 (0.10, 0.40)	0.30 (0.10, 0.45)	<0.001
	Morphine	0.11 (0.04, 0.17)	0.07 (0.04, 0.13)	0.17 (0.08, 0.67)	0.25 (0.15, 0.27)	<0.001
	Oxycodone ER	0.05 (0.04, 0.11)	0.06 (0.02, 0.08)	0.17 (0.11, 0.33)	0.08 (0.04, 0.21)	0.036

Abbreviations: MEDD: morphine equivalent daily dose. IQR: Inter quartile range. ER: Extended release. Ratio breakthrough/schedule MEDD per medication; data for the two most common prescribed breakthrough medications. * Continuous variables examined by Wilcoxon signed- rank-sum test.

## Data Availability

Data available on request: syennu@mdanderson.org.
